# Preoperative Sedentary Time Predicts Postoperative Complications in Gastrointestinal Cancer

**DOI:** 10.31557/APJCP.2020.21.11.3405

**Published:** 2020-11

**Authors:** Takuya Yanagisawa, Hideshi Sugiura, Noriatsu Tatematsu, Mioko Horiuchi, Saki Migitaka, Keita Itatsu

**Affiliations:** 1 *Department of Rehabilitation, Kamiiida Daiichi General Hospital, 2-70 Kamiiida-kitamachi, Kita-ku, Nagoya, Aichi 462-0802, Japan.*; 2 *Program in Physical and Occupational Therapy, Nagoya University Graduate School of Medicine, 1-1-20 Daiko-minami, Higashi-ku, Nagoya, Aichi 461-8673, Japan. *; 3 *Department of Integrated Health Sciences, Nagoya University Graduate School of Medicine, 1-1-20 Daiko-minami, Higashi-ku, Nagoya, Aichi 461-8673, Japan. *; 4 *Department of surgery, Kamiiida Daiichi General Hospital, 2-70 Kamiiida-kitamachi, Kita-ku, Nagoya, Aichi 462-0802, Japan. *

**Keywords:** Sedentary lifestyle, physical activity, postoperative complications, gastrointestinal cancer

## Abstract

**Background:**

Gastrointestinal cancer has a high global prevalence. Postoperative complications (PCs) affect the length of hospital stay and long-term outcomes. However, it is unclear whether preoperative sedentary time is associated with PCs, independently of physical activity (PA). We aimed to investigate the association between preoperative sedentary time and PCs independently of PA in patients who underwent surgery for gastrointestinal cancer.

**Methods:**

In this prospective study, we included 112 patients who underwent colorectal cancer or gastric cancer surgery. Patient characteristics and surgery-related variables were collected. The Japanese version of the International Physical Activity Questionnaire (the usual 7-day short version) was used to assess preoperative PA and sedentary time. Patients were classified into two groups according to the grade of PCs: Clavien-Dindo (CD) grade <2 and ≥2. Multivariate logistic regression analysis was performed to identify the risk factors for CD grade ≥2 PCs. Receiver operating characteristic curve analysis was used to determine the optimal cutoff point of sedentary time for predicting PCs with CD grade ≥2.

**Results:**

PCs occurred in 38 patients (33.9%). Sedentary time (odds ratio [OR] 1.29, 95% confidence interval [CI]: 1.09-1.53; p<0.01) and body mass index (OR 1.17, 95% CI: 1.01-1.36; p=0.03) were associated with PCs independently of total PA. The optimal cutoff point of sedentary time for predicting PCs was 6 h/day (sensitivity 0.662, specificity 0.658).

**Conclusion:**

Preoperative sedentary time is a predictor of PCs in patients who undergo gastrointestinal cancer surgery.

## Introduction

Globally, gastrointestinal cancers, such as colorectal and gastric cancers, are the most frequently diagnosed cancers in both sexes (Bray et al., 2018). Although advances in treatment techniques have improved survival rates in recent years, the incidence of postoperative complications (PCs) in cases with colorectal and gastric cancers is approximately 30% (Endo et al., 2017). Moreover, PCs affect the hospital length of stay (LOS) (Nakanishi et al., 2018; Zhang et al., 2018) and long-term outcomes, such as overall survival and overall recurrence rate (Law et al., 2007; Li et al., 2018). PCs in gastrointestinal cancer can be predicted by preoperative sarcopenia (Yang et al., 2019), frailty (Vermillion et al., 2017), 6-minute walk distance (6MWD) (Hayashi et al., 2017), fatigue (Heldens et al., 2017), and prognostic nutrition index (PNI) (Kanda et al., 2016). Thus, it is important to clinically assess modifiable factors to prevent PCs. 

Physical activity (PA) is an important factor that influences sarcopenia (Cruz-Jentoft et al., 2019) and frailty (Xue et al., 2008). However, while preoperative PA was reported to be associated with PCs in patients with colorectal cancer (Onerup et al., 2019), no such association was observed in another study (Heldens et al., 2017). There are very few reports examining the relationship between preoperative PA and PCs, as most studies have not investigated the intensity of PA. Thus, there is still no consensus on the relationship between preoperative PA and PCs in gastrointestinal cancer.

Sedentary time is one of the PA indicators and is defined as any waking behavior characterized by an energy expenditure of ≤1.5 metabolic equivalents (METs) while in a sitting or reclining posture (Barnes et al., 2012). Sedentary time has been shown to account for approximately 50% of daily living activities among community-dwelling people (Chen et al., 2018). A Previous meta-analysis indicated associations between sedentary time and all-cause mortality, cancer mortality, and cancer incidence, independently of PA (Biswas et al., 2015). Another meta-analysis reported that the mortality risk associated with increased sedentary time could only be eliminated by high levels of moderate-intensity PA (Ekelund et al., 2016). These reports suggest the importance of reducing sedentary time in order to prevent adverse outcomes. Moreover, reducing sedentary time rather than encouraging high level PA may be an easier task for patients to handle. Sedentary time is also reportedly associated with sarcopenia (Gianoudis et al., 2015) and frailty (Blodgett et al., 2015), which are risk factors for PCs independently of PA. Preoperative sedentary time is reportedly longer in esophageal cancer patients with than without pulmonary PCs (Feeney et al., 2011). However, the possibility of an association between preoperative sedentary time and PCs independently of PA in gastrointestinal cancer remains unclear. 

In the present study, we aimed to investigate the association between preoperative sedentary time and PCs independently of PA in patients with gastrointestinal cancer.

## Materials and Methods

*Patients*

In this prospective study, we enrolled 112 patients who underwent open or laparoscopic surgery for primary colorectal (n=81) or gastric cancer (n=31) between October 2016 and December 2019 at Kamiiida Daiichi General Hospital. Exclusion criteria were patients who (1) could not walk unassisted, (2) had cognitive dysfunction, (3) had simultaneous cancer, (4) underwent palliative surgery, and (5) had missing data. This study was approved by the Ethics Committee of Kamiiida Daiichi General Hospital and the Human Research Ethics Committee of the Nagoya University School of Health Sciences. Prior to participation in this study, all patients were provided with a thorough explanation about the study and provided written consent in accordance with the Declaration of Helsinki.

*Outcome*

The study outcome was the occurrence of PCs within 30 days after surgery. Clavien-Dindo (CD) classification was used to grade PCs (grade 1-5) (Dindo et al., 2004). To eliminate the possibility of description bias in patient records, grade 1 complications were excluded, and complications above CD grade 2 were considered as PCs.

*Preoperative physical activity and sedentary time*

Preoperative PA and sedentary time were assessed using the Japanese version of the usual 7-day short version of the International Physical Activity Questionnaire (IPAQ-SV). This questionnaire is used to assess vigorous- to moderate-intensity PA and walking activity during 7 usual days, and sedentary time during the usual a weekday (Craig et al., 2003). Each activity type and intensity score are assigned a METs value according to a published protocol (Sjostrom et al., 2005).

*Preoperative physical function, and fatigue*

To assess the physical function, we measured grip strength, usual gait speed, and 6MWD. Grip strength was measured once on each side using a dynamometer (Grip-D, TKK 5401; Takei Scientific Instruments Co., Niigata, Japan), and the average of each pair of measurements was calculated. Usual gait speed was measured over a 10-m distance between the 3- and 13-m marks of a 16-m walkway. To measure the 6MWD, we instructed patients to walk a predetermined course as far as possible for 6 minutes. The distance covered (in meters) by the patients in 6 minutes was described as the 6MWD. 

Fatigue was assessed using the Numerical Rating Scale (0, absence of fatigue – 10, maximum fatigue).

*Patient characteristics, surgery-related variables, and data collection*

Age, gender, body mass index (BMI), Brinkman index, presence of polypharmacy, pulmonary function, presence of diabetes mellitus (DM), hypertension, cerebrovascular disease, chronic obstructive pulmonary disease, and heart disease, Eastern Cooperative Oncology Group performance status, cancer type (colorectal or gastric), and pathological TNM stage (p-stage) were recorded as patient characteristics. Polypharmacy was defined as more than or equal to five daily medications and identified as the predictive factor of PCs (Volakis et al., 2018).

Surgery-related variables including surgical approach (open or laparoscopic), combined resection, operative time, blood loss, and postoperative hospital LOS were recorded. 

The preoperative serum levels of albumin, C-reactive protein (CRP), and hemoglobin, white blood cell count, and total lymphocyte count were collected from electronic medical records. The PNI, a nutritional status indicator, was assessed using the equation: PNI = 10 **×** serum albumin (mg/dL) + 0.005 **×** total lymphocyte count (Kanda et al., 2016).

*Statistical analysis*

All continuous variables were expressed as median (interquartile ranges). Patients were divided into the following two groups according to the grade of PCs: CD grade <2 and ≥2. Intergroup differences were analyzed using the *χ*^2^ test or Fisher’s exact test for categorical variables and the Mann-Whitney U test for continuous variables. Multivariate logistic regression analysis was performed to identify the risk factors for developing PCs of CD grade ≥2. We used the variables with p<0.05 as independent variables in this analysis. Multivariate logistic regression analysis was performed with (model 2) and without (model 1) total PA adjustment. Finally, receiver operating characteristic (ROC) curve analysis was performed to determine the optimal cutoff point of sedentary time. All statistical analyses were performed using EZR version 1.40 (Saitama Medical Center, Jichi Medical University, Saitama, Japan) (Kanda, 2013).

## Results

PCs classified as CD ≥2 occurred in 38 patients (33.9%). The most frequently observed PC was ileus (28.9%), followed by anastomotic leakage and drain retrograde infection (18.4% each) (Supplementary Table 1). 

Patient characteristics and comparisons of measured variables between the CD <2 and CD ≥2 groups are shown in Table 1. No significant differences were observed between the two groups, except for BMI, sedentary time, operative time, and postoperative hospital LOS. BMI was significantly higher in patients with CD ≥2 than in those with CD <2 group (p<0.01). Preoperative sedentary time, operative time, and postoperative hospital LOS were significantly longer in patients with CD ≥2 than in those with CD <2 (p<0.01, p=0.01, and p<0.01, respectively). Combined resection sites were mostly in the gall bladder (Supplemental Table 2).

The results of multivariate logistic regression analysis are shown in Table 2. Sedentary time, BMI, and operative time, all had a value of p<0.05 on univariate analysis, and therefore, underwent multivariate analysis as potential risk factors for PCs. Sedentary time and BMI were identified as risk factors for developing PCs with CD ≥2 independently of total PA. The respective odds ratios for the occurrence of PCs with CD ≥2 were 1.29 (95% confidence interval [CI]: 1.09-1.53; p<0.01) and 1.17 (95%CI: 1.01-1.36; p=0.03) in increments of 1 h/day for sedentary time and 1 kg/m^2^ for BMI (Table 2).

**Figure 1:**
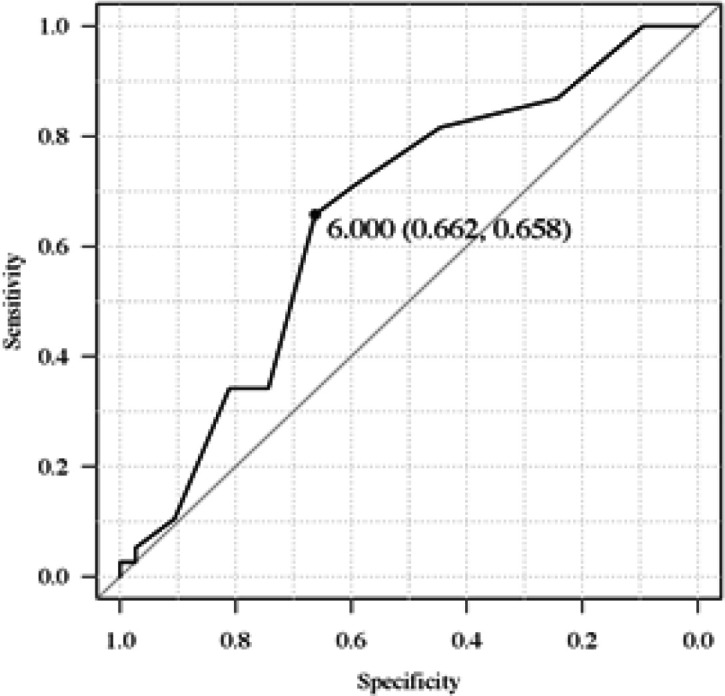
The Receiver Operating Characteristic Curve of Preoperative Sedentary Time for Predicting PCs with CD Grade ≥2. The area under the curve was 0.65 (95% CI: 0.55-0.75).

The area under the ROC curve for preoperative sedentary time was 0.65 (95% CI: 0.55-0.75; p<0.01). The optimal cutoff point of preoperative sedentary time for predicting PCs with CD grade ≥2 was 6 h/day (sensitivity 0.662, specificity 0.658) (Figure 1). 

Patient characteristics and comparisons of measured variables between patients with sedentary time <6 h/day and those with sedentary time ≥6 h/day are shown in Table 3. Brinkman index and CRP were significantly higher in patients with sedentary time ≥6 h/day compared to those with sedentary time <6 h/day (p=0.02 and p=0.03, respectively). Significantly higher numbers of patients with sedentary time ≥6 h/day were exposed polypharmacy, had DM, and developed PCs with CD grade ≥2 compared to those with sedentary time <6 h/day (p=0.02, p<0.01, and p<0.01, respectively). Total PA, vigorous-intensity activity, moderate-intensity activity, and walking activity were significantly fewer in patients with sedentary time ≥6 h/day than those with sedentary time <6 h/day (p<0.01, p=0.03, p=0.01, and p<0.01, respectively).

**Table 1:** Univariate Analysis of Patient Characteristics and Measured Variables in Patients Having PCs with CD Grade <2 and ≥2

	CD <2 (n = 74)	CD ≥2 (n = 38)	p - value
Age, years	71 (64-77)	71 (63-80)	0.62
Gender, n (male/female)	44 / 30	27 / 11	0.27
BMI, kg/m^2^	22.0 (19.5-24.6)	24.3 (21.6-25.4)	<0.01
Brinkman index	345 (0-620)	425 (0-762)	0.6
Polypharmacy, n (yes/no)	30 / 44	22 / 16	0.08
%VC, %	96.3 (87.3-108.7)	93.9 (85.0-106.7)	0.37
FEV1.0%, %	78.3 (73.5-82.8)	78.5 (72.9-83.5)	0.99
PS, n (0/1)	55 / 19	32 / 6	0.23
DM, n (yes/no)	17 / 57	15 / 23	0.06
HT, n (yes/no)	37 / 37	20 / 18	0.79
CVD, n (yes/no)	66 / 8	35 / 3	0.74
COPD, n (yes/no)	72 / 2	37 / 1	0.99<
HD, n (yes/no)	70 / 4	35 / 3	0.68
Cancer type, n (colorectal/gastric)	56 / 18	25 / 13	0.26
P-stage, n (0^a^)	50 / 24	24 / 14	0.64
PNI	48.1 (43.8-53.3)	49.2 (45.1-53.1)	0.6
Albumin, g/dL	4.0 (3.7-4.3)	4.0 (3.6-4.3)	0.87
CRP, mg/dL	0.17 (0.07-0.48)	0.32 (0.13-0.57)	0.07
Hemoglobin, g/dL	12.6 (11.3-14.4)	13.1 (11.2-14.8)	0.53
WBC, ×10^3^/μL	6.3 (5.3-7.9)	6.5 (5.3-7.3)	0.8
TLC, ×10^3^/μL	1.6 (1.2-2.0)	1.7 (1.4-2.1)	0.12
Total PA, METs-h/week	1270 (282-2115)	1386 (693-2970)	0.26
Vigorous-intensity activity, METs-h/week	0 (0-0)	0 (0-0)	0.49
Moderate-intensity activity, METs-h/week	0 (0-240)	0 (0-15)	0.53
Walking activity, METs-h/week	1270 (282-2115)	1386 (693-2970)	0.41
Sedentary time, h/day	4 (2.75-7)	6 (4-8)	<0.01
Grip strength, kg	25.4 (19.9-32.6)	30.9 (21.5-35.5)	0.12
Usual gait speed, m/s	1.22 (0.97-1.43)	1.32 (1.13-1.49)	0.1
6MWD, m	467 (368-531)	460 (427-540)	0.36
Fatigue, points	0 (0-3)	0 (0-1)	0.47
Surgical approach, n (open/laparoscopy)	40 / 34	17 / 21	0.35
Combined resection, n (yes/no)	12 / 62	9 / 29	0.33
Operative time, min	245 (189-313)	287 (229-407)	0.01
Blood loss, mL	89 (28-352)	142 (39-322)	0.16
Postoperative hospital LOS, days	11.5 (10-14)	21.5 (16-32)	<0.01

a, colorectal cancer only; CD, Clavien-Dindo; PCs, postoperative complications; BMI, body mass index; %VC, % vital capacity; FEV1.0%, forced expiratory volume in 1 second %; PS, performance status; DM, diabetes mellitus; HT, hypertension; CVD, cerebrovascular disease; COPD, chronic obstructive pulmonary disease; HD, heart disease; P-stage, pathological stage; PNI, prognostic nutrition index; CRP, C-reactive protein; WBC, white blood cell count; TLC, total lymphocyte count; PA, physical activity; METs, metabolic equivalents; 6MWD, 6-minute walk distance; LOS, length of stay

**Table 2:** Multivariate Logistic Regression Analysis for the Risk of PCs with CD Grade ≥2

	Model 1		Model 2	
	OR (95% CI)	P-value	OR (95% CI)	P-value
Sedentary time, per 1 h/day	1.21 (1.05-1.40)	<0.01	1.29 (1.09-1.53)	<0.01
BMI, per 1 kg/m^2^	1.15 (1.00-1.34)	0.05	1.17 (1.01-1.36)	0.03
Operative time, per 1 min	1.00 (0.99-1.00)	0.09	1.00 (0.99-1.00)	0.2

Three variables with p<0.05 (i.e., sedentary time, BMI, and operative time) in univariate analysis were entered into the multivariate logistic regression models; Model 2 was adjusted for total PA; CD, Clavien-Dindo; PCs, postoperative complications; OR, odds ratio; CI, confidence interval; BMI, body mass index; PA, physical activity

**Table 3:** Univariate Analysis of Patient Characteristics and Measured Variables in Patients with Sedentary Time <6 h/day and ≥6 h/day

	Sedentary time <6 h/day (n = 62)	Sedentary time ≥6 h/day (n = 50)	p value
Age, years	68.5 (63-76.25)	74 (66.75-79.25)	0.05
Gender, n (male/female)	37 / 25	34 / 16	0.36
BMI, kg/m^2^	23.1 (20.0-25.3)	22.7 (19.9-24.9)	0.71
Brinkman index	200 (0-570)	500 (0-800)	0.02
Polypharmacy, n (yes/no)	23 / 39	29 / 21	0.02
%VC, %	96.3 (86.7-109.3)	94.8 (86.7-106.9)	0.81
FEV1.0%, %	78.3 (73.8-83.6)	78.6 (71.6-82.6)	0.81
PS, n (0/1)	48 / 14	39 / 11	0.94
DM, n (yes/no)	Nov-51	21 / 29	<0.01
HT, n (yes/no)	28 / 34	29 / 21	0.17
CVD, n (yes/no)	56 / 6	45 / 5	0.99<
COPD, n (yes/no)	61 / 1	48 / 2	0.58
HD, n (yes/no)	59 / 3	46 / 4	0.69
Cancer type, n (colorectal/gastric)	49 / 13	32 / 18	0.07
P-stage, n (0^a^)	43 / 19	31 / 19	0.41
PNI	47.6 (43.4-53.0)	49.2 (46.0-53.4)	0.16
Albumin, g/dL	4.0 (3.6-4.3)	4.0 (3.8-4.3)	0.54
CRP, mg/dL	0.15 (0.07-0.48)	0.31 (0.13-0.50)	0.03
Hemoglobin, g/dL	12.5 (11.3-14.5)	13.0 (11.1-14.9)	0.68
WBC, ×10^3^/μL	6.2 (5.3-7.3)	6.7 (5.3-8.0)	0.41
TLC, ×10^3^/μL	1.6 (1.3-1.9)	1.8 (1.3-2.3)	0.24
Total PA, METs-h/week	1827 (855-3386)	763 (0-1386)	<0.01
Vigorous-intensity activity, METs-h/week	0 (0-0)	0 (0-0)	0.03
Moderate-intensity activity, METs-h/week	0 (0-480)	0 (0-0)	0.01
Walking activity, METs-h/week	1386 (684-2772)	459 (0-1386)	<0.01
Grip strength, kg	27.8 (19.0-34.4)	27.3 (20.7-33.4)	0.97
Usual gait speed, m/s	1.28 (1.04-1.45)	1.18 (1.00-1.39)	0.15
6MWD, m	475 (380-546)	452 (373-502)	0.32
Fatigue, points	0 (0-1.5)	0 (0-2)	0.71
Surgical approach, n (open/laparoscopy)	28 / 34	29 / 21	0.17
Combined resection, n (yes/no)	11 / 51	10 / 40	0.76
Operative time, min	271 (195-365)	253 (206-339)	0.72
Blood loss, mL	100 (36-319)	115 (27-350)	0.78
PCs, n (yes/no)	13 / 49	25 / 25	<0.01
Postoperative hospital LOS, days	12 (10-18)	14.5 (10-22)	0.4

a, colorectal cancer only; BMI, body mass index; %VC, % vital capacity; FEV1.0%, forced expiratory volume in 1 second %; PS, performance status; DM, diabetes mellitus; HT, hypertension; CVD, cerebrovascular disease; COPD, chronic obstructive pulmonary disease; HD, heart disease; P-stage, pathological stage; PNI, prognostic nutrition index; CRP, C-reactive protein; WBC, white blood cell count; TLC, total lymphocyte count; PA, physical activity; METs, metabolic equivalents; 6MWD, 6-minute walk distance; PCs, postoperative complications; LOS, length of stay.

## Discussion

The present study demonstrated that PCs with CD grade ≥2 in patients who underwent gastrointestinal cancer surgery could be predicted by preoperative sedentary time independently of total PA. Thus, preoperative sedentary time might be a useful screening tool to identify patients at a high risk of PCs with CD grade ≥2 following gastrointestinal cancer surgery. To our knowledge, the present study is the first to reveal the relationship between preoperative sedentary time and PCs, independently of total PA, in patients who underwent gastrointestinal cancer surgery. 

In the present study, Brinkman index, CRP, and the number of patients exposed to polypharmacy and had DM were significantly higher among patients with sedentary time ≥6 h/day compared to those with sedentary time <6 h/day. In previous studies, sedentary time, as assessed by a questionnaire, was found to be associated with insulin resistance, inflammatory markers, and the number of medications (Celis-Morales et al., 2012; Yates et al., 2012; Heseltine et al., 2015). Moreover, the preoperative Brinkman index, polypharmacy, DM, and CRP were associated with PCs (Frisch et al., 2010; Kubo et al., 2013; Xue et al., 2018; Yoshikawa et al., 2019). Previous experimental studies have indicated that even short-term sedentary behavior affects systemic function. Restaino et al., (2015) reported that sitting for 6 hours markedly reduced lower leg micro- and macrovascular dilator function in young healthy men. Hamburg et al., (2007) reported that 5-day bed rest increased insulin resistance, serum glucose, total cholesterol, and systolic blood pressure, and decreased calf blood flow in healthy volunteers. It was speculated that prolonged sedentary time affected the systemic functions and increased the risk of PCs; however, the detailed mechanisms are unknown and require further study. 

In the present study, the optimal cutoff point of preoperative sedentary time for predicting PCs with CD grade ≥2 was 6 h/day, with a sensitivity of 66.2% and specificity of 65.8%. One previous study reported that individuals with sedentary time ≥6 h/day had a higher risk of metabolic syndrome than those with sedentary time <6 h/day (Petersen et al., 2014). Another study showed that individuals with sedentary time >5 h/day had higher all-cause mortality than those with sedentary time ≤5 h/day (Larsson and Wolk, 2019). Thus, we believe that our cutoff value (6 h/day) is clinically significant in predicting PCs notwithstanding its low sensitivity and specificity. According to Coqueiro (2017), the cutoff point of sedentary time as assessed by IPAQ-SV was 7 h/day for predicting frailty; according to Ohashi (2018), that for predicting sarcopenia was 8 h/day. The optimal cutoff value may vary depending on the patient characteristics and outcomes; therefore, further research is warranted.

We also revealed an association between BMI and PCs with CD grade ≥2. A previous meta-analysis reported that BMI was associated with PCs in patients with colorectal (Almasaudi et al., 2018) and gastric cancer (Zhao et al., 2018); which corroborates with our study results. In previous studies, operative time and blood loss were significantly higher in high than in low BMI patients (Hede et al., 2015; Feng et al., 2018), suggesting that a high BMI might predispose patients to more surgical stress.

In the present study, sedentary time was associated with PCs, unlike preoperative PA, grip strength, usual gait speed, 6MWD, PNI, and fatigue. This finding differed from those of previous studies (Kanda et al., 2016; Hayashi et al., 2017; Heldens et al., 2017; Onerup et al., 2019). Possibly, that physical functioning and nutritional status may not directly reflect more sedentary time and a lower level of PA. The results of the present study may suggest the importance of reducing sedentary time rather than exercise intervention to prevent PCs in patients without sarcopenia or frailty, who have no or minimal physical function or nutritional status decline.

Our study has several limitations. First, the results may not adequately adjust for confounding factors due to a small number of patient sample size. Thus, our results must be interpreted with caution. Second, the present study included patients who underwent colorectal surgery and gastric surgery. Further studies are necessary to separately analyze patients with colorectal and gastric cancer. Finally, it was not possible to study the different types of PCs because of the small patient sample size. It is necessary to conduct further large-scale studies to analyze PCs by type.

In conclusion, preoperative sedentary time could predict PCs in patients who underwent surgery for gastrointestinal cancer.
